# Polygamy, sexual behavior in a population under risk for prostate cancer diagnostic: an observational study from the Black Sea Region in Turkey

**DOI:** 10.1590/S1677-5538.IBJU.2017.0525

**Published:** 2018

**Authors:** Abdullah Cirakoglu, Erdal Benli, Ahmet Yuce

**Affiliations:** 1 Department of Urology, Ordu University, Faculty of Medicine, Ordu, Turkey

**Keywords:** Prostatic Neoplasms, Marriage, Sexual Behavior

## Abstract

**Aim:**

Although prostate cancer (PCa) is the most common cancer type in men, a replaceable risk factor has not yet been established. In our study, we assessed the relationship between the number of sexual partners, age of first sexual experience and age of first masturbation and prostate cancer incidence.

**Materials and Methods:**

In Ordu University Department of Urology between January 2013 and September 2016, in PSA elevation and rectal examination, patients with prostate biopsy were evaluated due to nodule palpation in the prostate. At younger ages and at present, their first masturbation ages, first sexual debut ages, and total sexual partner numbers were recorded. The correlation between the obtained data and PCa frequency was evaluated.

**Results:**

The study included 146 patients with PCa identified on biopsy and 171 patients with benign biopsy results who answered the questions. 66.7% of the ones whose biopsy results were benign and 40.6% of cancer suspects had only one sexual partner. The median number of sexual partners was 1±4 (1-100) in the benign group and 2±6 (1-500) in the malignant group (p=0.039). There was a negative correlation between age of first sexual debut and number of partners (r: −0,479; p <0.001).

**Conclusion:**

In our study, it appears that there may be an association between the number of sexual partners and prostate cancer in the patient group with PSA level above 4ng/mL. Avoidance of sexual promiscuity or participation in protected sex may be beneficial to protect against prostate cancer.

## INTRODUCTION

Prostate cancer (PCa) is the most common cancer type in males and in second place in terms of cancer-linked deaths (1). Though this is a very common cancer, the etiology is only partly understood. Age, ethnicity and family history are known to be important for the etiology (2). Studies of migrants have reported environmental factors and lifestyle factors may be important (3). However, the majority of these factors cannot be changed. A variable risk factor for PCa has still not been definitely determined. Studies have related PCa risk with factors linked to more active sexual life (4). Sexual activity include various dimensions like sex of the partners, number of sexual partners, frequency of ejaculation and age of first sexual debut. It is thought that all of these may affect PCa development in negative or positive ways to varying degrees. Some studies have reported that sexual behavior and sexually transmitted disease may play a role in the etiology of prostate cancer (5). In our study, we aimed to assess the correlation between number of sexual partners, monthly frequency of sexual relations, age of first sexual debut and age of first masturbation with the incidence of prostate cancer.

## MATERIALS AND METHODS

Patients undergoing prostate biopsy at Ordu University Medical Faculty Urology Clinic from January 2013 to September 2016 were assessed. Patients with PSA>4 or nodule felt during digital rectal examination were referred for biopsy. All biopsies were completed in 12 quadrants with TRUS guidance. All patients provided information to a doctor during an interview. Informed consent forms have been obtained as the patients have been informed that their data would be kept confidential and used for research purposes. The answers to questions about frequency of sexual relations when young and currently, age of first masturbation, age of first sexual experience and total numbers of sexual partners were recorded. The correlation of the obtained data with the incidence of PCa was assessed. Patients who did not wish to answer the questions were excluded from the study. The study included 146 patients with PCa identified on biopsy and 171 patients with benign biopsy results who answered the questions. Patients lived in the same geographical region, and had similar lifestyle and nutritional habits. All males in our study group were circumcised. Our study is an observational study. This study was approved by Ordu Univercity Local Ethic Comity (2017/167).

### Statistical analysis

Data were tested for normal distribution with the Kolmogorov-Smirnov test. Data with normal distribution were compared with the independent groups t test (student t), whi le data with non-normal distribution were compared with the Mann-Whitney U test. The Spearman correlation coefficient was used to assess the correlations among groups with normal distribution. Results with p<0.05 were accepted as statistically significant.

## RESULTS

Of 1072 patients with PSA values on record, 317 patients with prostate biopsy completed and necessary information available were assessed. Of these patients, 171 had benign biopsy results, while 146 patients had PCa identified. The mean age of patients in the benign group was 64.38±8.20 (41-92) years and in the malignant group was 67.39±9.34 (43-97) years. The median PSA values were 6.55±4.86 (2.28-42.00) in the benign group and 8.60±13.15 (3.26-934.30) in the malignant group. In terms of sexual partners, 66.7% of the benign group and 40.6% of the malignant group had only one sexual partner. Median number of sexual partners was 1±4 (1-100) in the benign group and 2±6 (1-500) in the malignant group. The difference in numbers of sexual partners was found to be statistically significant between the groups (p=0.039). There was no statistically significant difference between the two groups in terms of number of relations per month when young and currently, first masturbation age and first sexual debut age. Details of data and p values are shown in Table-1. There was a negative correlation identified between age of first sexual debut and number of partners (r: −0.479; p<0.001).

**Table 1:** Distribution of data according to malignant and benign groups.

Variables	Groups		P-value
	Benign	Malign	
Patients age	63.03±8.07^a^	66.24±7.73^a^	0,002**
Psa (ng/dL)	6.40±3.83 (3.60-25.20)^b^	8.6±10.16 (0-330)^b^	0.001**
Number of sexual partners	1±4 (1-100)^b^	2±6 (1-500)^b^	0.039*
Monthly sexual intercourse frequency in youth	10±7 (3-30)^b^	10±6 (2-24)^b^	0.236
Current monthly sexual intercourse frequency	4±5 (0-22)^b^	4±6 (0-17)^b^	0.957
First masturbation age	15±15 (0-18)^b^	12±16 (0-35)^b^	0.018*
Age of first sexual debut	20±6 (14-27)^b^	18±6 (13-52)^b^	0.137

a = mean±SD;

b = median±IQR (min-max);

* = p<0.05);

** = p<0.01

## DISCUSSION

Our study found an association between the number of sexual partners over life and positive biopsy for PCa among patients at risk of PCa diagnosis. It is thought that sexually transmitted infections (STI) are a significant factor for the link with PCa. A study by Rosenblatt et al. identified a positive correlation between the number of female sexual partners and PCa risk (6). Studies related to this topic have found that syphilis identified by serologic tests increases the risk of prostate cancer by 1.8 times and this increase is correlated with the number of sexual partners (7). Two meta-analyses published in 2002 and 2005 identified that sexually transmitted infections increased the risk of PCa (4, 8). It is thought that factors like bacteria and fungus causing infections and injury to the prostate stimulate inflammasome-media-ted pro inflammatory cytokines beginning tumor progression (9). The role of inflammation in PCa development has been known for a long time. It is thought that inflammation encourages angiogenesis and DNA damage causing carcinogenesis (10-12). A PCPT study showed that inflammation observed in prostate biopsy specimens is related to high grade cancer developing in advanced periods (13). Proinflammatory cytokines and chemokines released from immune cells aid the formation of neoplastic cells (9). In our study, we believe the relationship between number of partners and incidence of PCa may be due to sexually transmitted infections and prostatic inflammation linked to these infections.

There are studies showing that age of initial sexual activity and frequency of ejaculation are effective in development of PCa. The increase in frequency of ejaculation reduces the carcinogenic material concentration within prostatic fluids and intraluminal prostatic crystalloid accumulation and thus it is reported that frequency of ejaculation may be protective against cancer (14-16). In our study, we identified that both groups were similar in terms of the markers of ejaculation frequency of first masturbation age, first sexual debut age and frequency of sexual relations when young and currently. Among data assessing sexual life, a significant difference was only found for the number of sexual partners. The median number of sexual partners and the number of those with more than one sexual partner in the malignant group were identified to be statistically significantly higher compared to the benign group.

In our study, we identified that as the age of first sexual debut reduced, the number of partners increased. There are different interpretations in the literature related to the effect of age of first sexual debut and number of partners on PCa development. Rotkin et al. reported that early age of first sexual debut increased the risk of prostate cancer (17). Rosenblatt et al. proposed that rather than an early start to sexual relations, the number of partners was important (6). In fact, the study by Rotkin did not assess the effect of number of sexual partners. Ahmadi et al. reported that the risk of prostate cancer was lower for males who married at a young age (18). The study by Ahmadi was based in Iran. Iranian Muslims are explicitly different to Western societies as a result of tight governmental restrictions on open sexual relationships and prostitution as well as cultural and religious fixation on avoiding premarital and extramarital sexual activity. In our study, as the age of sexual debut reduced, the number of partners increased and we found the number of partners was related to incidence of prostate cancer. When all this data is assessed with our results, it appears that the number of sexual partners is important for PCa development.

Our study group comprises a homogeneous group living in a small geographical area. The study region does not have inward migration and is a relatively small area. As the region is small, it may not be possible to generalize our results to the whole population. However, it is considered that genetic factors, nutritional habits and environmental factors are effective in the etiology of PCa. An advantage of our study is that the study group comprises people living in the same geographic region, with similar life styles, nutritional forms and genetic characteristics. In both our patient groups, the effects of these factors may be accepted as being similar. Additionally, in our country, society avoids open conversation related to sexuality. It is very difficult to obtain information about previous sexual experience from males of adult age. There is a risk that information given on the question forms and in interviews is deficient or wrong. In our clinic, a single doctor works with patients to resolve concerns of patients worried due to the possibility of prostate cancer and to ensure compliance with testing and follow-up during this process. More time is allotted to patients and confidence is given that the information that they provide will remain confidential. The diagnosis, treatment and process after treatment for these patients is performed by the same doctor. Due to the trust built up in this environment, we believe the information given is accurate. Thus, it is very hard to record data for this number of patients from a small region. As a result, our study is the first study to investigate this data in Turkey.

There are some limitations of our study. The first is that serologic testing was not completed to assess our patients for STI. As a result, we do not have clear results in terms of previous STI history. However, as the number of partners increase, an increase in incidence of STI is expected. Studies have shown that experiencing STI clearly increases the chances of PCa. In our study, as the number of sexual partners increased, there was an increase observed in the incidence of prostate cancer. Another limitation is that though biopsy was benign in the group with high PSA, there is a chance of occurrence on 2nd and 3rd biopsies. Although, the number of patients with a single sexual partner was higher in the group with benign biopsy results, there was a considerable number of patients with multiple sexual partners in both groups. As the size of groups was limited in our study, further studies with larger sample size can give different results. Since both of the groups consisted of patients with high PSA values, some of the patients with benign biopsy result may develop prostate cancer later in their life and diagnosed by a second or third biopsy. Therefore, it is better to say “there was a significant relationship between number of sexual partners and prostate cancer detection by first biopsy”. We believe it will be beneficial to perform a similar comparison with a control group with low PSA levels and hence a low risk of PCa.

## CONCLUSIONS

In our study, it appears that there may be an association between the number of sexual partners and prostate cancer in the patient group with PSA level above 4ng/mL. There was no association found between frequency of sexual relations in youth and at present, first age of masturbation and first age of sexual experience with PCa. Though not clearly revealed by our study, when the literature and our results are assessed together, we hypothesize that avoidance of sexual promiscuity or participation in protected sex may be beneficial to protect against prostate cancer. However, there is a need for more comprehensive studies to confirm this.
